# Optimization of medium parameters by response surface methodology (RSM) for enhanced production of cutinase from *Aspergillus* sp. RL2Ct

**DOI:** 10.1007/s13205-016-0460-4

**Published:** 2016-06-29

**Authors:** Vijaya Kumari, Vijay Kumar, Ravin Chauhan, Mohammad Asif, Tek Chand Bhalla

**Affiliations:** Department of Biotechnology, Himachal Pradesh University, Summer Hill, Shimla, 171005 Himachal Pradesh India

**Keywords:** Cutinase, *Aspergillus* sp. RL2Ct, Plackett–Burman design, Response surface methodology

## Abstract

Cutinases are hydrolytic enzymes which catalyzes esterification and trans-esterification reactions that make them highly potential industrial biocatalyst. In the present investigation microorganisms showing cutinase activity were isolated from plant samples. The strain showing maximum cutinase activity was identified by 18S rDNA sequencing as *Aspergillus* sp. RL2Ct and was selected for further studies. To achieve maximum enzyme production, the medium components affecting cutinase production were screened by Plackett–Burman followed by central composite design. The results obtained suggested that cutin, temperature and CaCl_2_ have influenced the cutinase production significantly with very high confidence levels. Cutinase production was maximum (663 U/mg protein) when using cutin prepared from orange peel as sole source of carbon. An overall 4.33-fold increase in the production of cutinase was observed after optimization of culture conditions (including 2.5-fold increase using RSM) during 24 h of incubation. The production time of *Aspergillus* sp. RL2Ct cutinase is significantly lower than the most of the earlier reported cutinase-producing fungus.

## Introduction

The cuticle protects the leaves, fruits and other softer parts of plant from dehydration and it acts as the outermost barrier in plant parts to infestation by pathogens. The major constituent of the cuticle is insoluble lipid polyester called cutin, which primarily consists of hydroxylated 16- and 18-carbon fatty acids that are linked together via ester bonds. Cutinase/cutin hydrolase (3.1.1.74) secreted by phytopathogenic microorganisms are inducible in nature and are capable of degrading cutin polymers of plant cell wall (Purdy and Kolattukudy 1975). These enzymes are widely distributed in animals, plants and microorganisms. Cutinases have attracted attention of researchers because of the fact that they do not require any cofactors, are fairly stable, catalyzes both hydrolysis and synthetic reactions and are even active in organic solvents. These are versatile enzymes showing several interesting properties which have potential industrial applications (Macedo and Pio 2005). In the food industry, cutinases have been used as important catalyst to produce dehydrated fruits, dairy products, flavoring compounds and some important fatty acids such as eicosapentanoic acid (EPA) and docosahexanoic acid (DHA) (Dutta et al. 2009). These enzymes have been also used to degrade various insoluble polymer films like poly(ethylene terephthalate) (PETP) and poly(*ε*-caprolactone) (PCL) (Murphy et al. 1996; Liu et al. 2009; Korpecka et al. 2010; Greimel et al. 2013). X-ray crystallographic studies of *F. solani pisi* cutinase have revealed that a catalytic triad Ser-His-Asp is located in its active site (Egmond and Vlieg 2000). Fungal cutinases can hydrolyse not only cutin, but also insoluble triglycerides as well as soluble esters such as *p*-nitrophenyl butyrate (*p*-NPB). Thus, fungal cutinases are considered as being intermediate between lipases and esterases (Carvalho et al. 1999; Longhi and Cambillau 1999).

In the present investigation, optimization of culture conditions for enhanced production of cutinase from *Aspergillus* sp. RL2Ct isolated from plant material has been carried out.

## Materials and methods

### Chemicals

All the chemicals used were of analytical grade and were purchased from Merck and Hi-Media, India. The substrate analogues, i.e., *p*-nitrophenyl palmitate (*p*-NPP) and *p*-nitrophenyl butyrate (*p*-NPB) used for cutinase assay were procured from Sigma.

### Preparation of cutin

Ripe oranges, papayas, tomatoes, apples and cucumbers purchased from the local market were used to extract cutin. The fruits/vegetables were peeled and the peels were added to boiling oxalate buffer solution (0.4 % oxalic acid, 1.6 % ammonium oxalate in 3:4 ratio and pH was adjusted to 3.2) for 15 min or until these became fully devoid of pulp. The peels were filtered, washed and dried at 40 °C, extracted with chloroform–methanol and vacuum dried. Dried pellet was crushed and ground to powder form and treated with cellulase and pectinase (Macedo and Pio 2005). The fine powder was used as substrate (carbon source) in subsequent experiments.

### Isolation and screening of cutinase-producing fungi

#### Sample collection and isolation

Many plant samples (decaying/fungal infected fruits and leaves) were collected from different parts of Shimla, Himachal Pradesh, India. The samples were homogenized, serially diluted in the sterile physiological saline, spread on potato dextrose agar and was incubated at 30 °C for 48 h. The cutinase-producing fungal species were selected, purified through repeated subculture and used for further studies.

#### Identification of cutinase-producing organism

The cellular morphology of the fungal isolates was examined. Morphological identification and spore staining of fungal isolate RL2Ct were performed. Further the identification of fungus was confirmed by 18S rDNA sequencing and phylogenetic analysis was carried out. Sequence obtained from Xceleris laboratories, Ahmadabad, India, was analyzed using BLAST and ClustalW. Phylogenetic tree was constructed with 1000 bootstrap replicates by using MEGA version 5.2 software (Tamura et al. 2011).

#### Production of cutinase

An aliquot of 1 ml spore suspension containing 2.11 × 10^7^ spores of *Aspergillus* sp. RL2Ct was added to 20 ml of seed medium (potato dextrose broth) and incubated at 30 °C for 24 h at 130 rpm on a gyratory incubator shaker. Two percent (% v/v) of seed medium was used to inoculate production medium [containing (g/l) NaNO_3_ 3.0, K_2_HPO_4_ 1.0, KCl 0.2, FeSO_4_·7H_2_O 0.01 and cutin 1, pH 7.0] and incubated at 30 °C for 72 h at 100 rpm on gyratory incubator shaker. After centrifugation at 10,000 × g for 10 min at 4 °C, the supernatant was collected and termed as crude cutinase and its activity was assayed.

#### Cutinase assay

The cutinase activity was determined according to the procedure of Winkler and Stuckmann (1979), with slight modification. In brief, the stock solution of the substrate (20 mM of *p*-nitrophenyl butyrate) was prepared in ethanol. Reaction mixture comprising 5 mM of substrate, 20 µl of enzyme preparation and 2.9 ml of potassium phosphate buffer (pH 7.0), was incubated at 30 °C for 10 min. The release of *p*-nitrophenol in the reaction was measured spectrophotometrically at 405 nm against a control reaction. One unit of cutinase activity was defined as the amount of enzyme which convert 1 µmol of substrate into product per minute.

#### Protein estimation

Protein concentration was determined using the Bradford method (Bradford 1976). Bovine serum albumin was used as the protein standard to construct the calibration curve.

#### Selection of medium

*Aspergillus* sp. RL2Ct was cultivated in different media M1 (KH_2_PO_4_ 0.5 g, K_2_HPO_4_ 2.5 g, NaCl 0.1 g, MgSO_4_ 0.2 g, CaCl_2_ 0.06 g, FeSO_4_ 0.03 g, cutin 1 g/l), M2, M3, M4, M5 (Macedo and Pio 2005; Dantzig et al. 1986; Dutta et al. 2013; Fett et al. 1992) with slight modifications which were earlier used for the growth of microorganism and production of cutinase. The selection of medium components was done through traditional ‘one-factor-at-a-time’ approach.

#### Screening of carbon and nitrogen sources

**Fig. 1:**
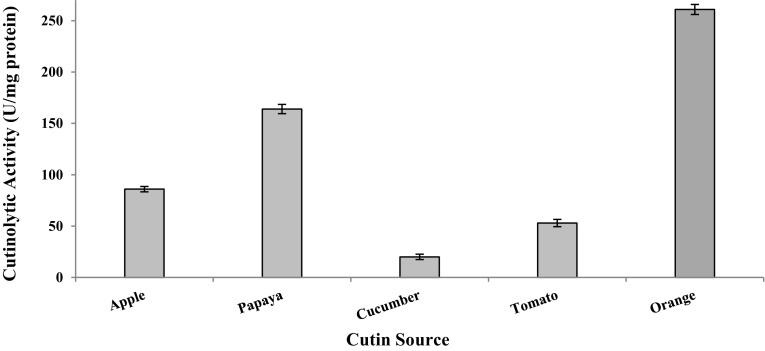
Effect of cutin (from various sources) on the cutinase production by *Aspergillus* sp. RL2Ct

Various carbon sources [1 % each of fructose, galactose, glucose, glycerol, lactose, maltose, mannitol, sucrose, cutin (tomato, apple, papaya, apple and orange)] and nitrogen sources (1 % each of yeast extract, beef extract, peptone, ammonium chloride, ammonium sulfate and urea) were screened to select suitable carbon and nitrogen source for the production of cutinase (Fig. 1).

#### Screening of important nutrients component using Plackett–Burman design (experimental design technique)

The medium components and physical culture parameters were screened for 11 variables. Each variable is represented at two levels, i.e., a high (+) and low (−). Eleven different variables considered in the present studies included pH, temperature, time, inoculum, K_2_HPO_4_, KH_2_PO_4_, NaCl, MgSO_4_, FeSO_4_, CaCl_2_ and cutin. Design Expert (Version 9.0) was used for Plackett–Burman design and regression analysis. The effect of each variable was calculated using the following equation:

$$E = \left(\sum {\text{M}}^{ + } - \sum {\text{M}}^{ - } \right)/N ,$$

where *E* is the effect of the tested variable and M^+^ and M^−^ are responses (enzyme activity) of trials at which the parameter was at its higher and lower level, respectively, and *N* is the number of experiment carried out. According to Plackett–Burman experimental design, a total of 12 experiments were performed. The levels of variables and design matrix in the coded levels and real values are shown in the Table 2.

#### Central composite design (CCD) and validation of statistical model

The variables which showed positive effect (cutin, temperature, CaCl_2_·2H_2_O) on the production of cutinase were optimized using central composite design (CCD). The minimum and maximum ranges of the selected variables, i.e., cutin 0–0.5 %, temperature 20–35 °C, CaCl_2_·2H_2_O 0.001–0.1 % were used in 20 combinations (Table 3). The statistical model was validated for the production of cutinase production by performing experiment in a shake flask under predicted set of conditions.

## Results

### Isolation and screening of cutinase-producing fungi

**Fig. 2:**
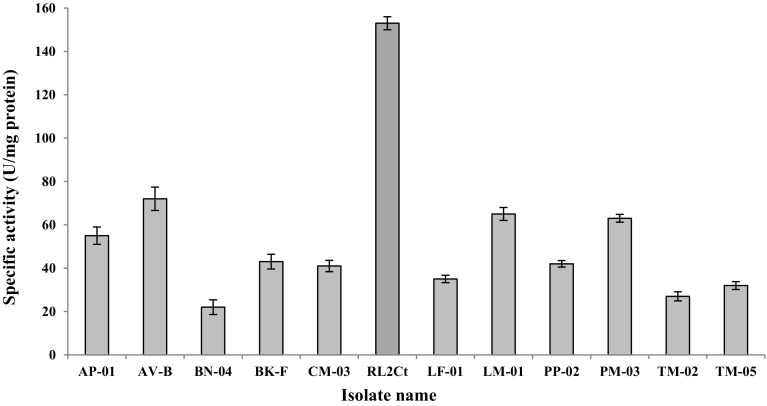
Cutinolytic activity of some fungal isolates

Sixty-nine fungal isolates were obtained from decaying/fungal infected plant samples, out of which only 12 isolates exhibited cutinase activity (Fig. 2) using minimal salt medium (pH 7.0) supplemented with 0.1 % cutin. The maximum cutinase activity was recorded in fungal isolate RL2Ct (153 ± 3.0 U/mg protein).

### Identification of fungal isolate RL2Ct by 18S rDNA analysis and phylogenetic study

**Fig. 3:**
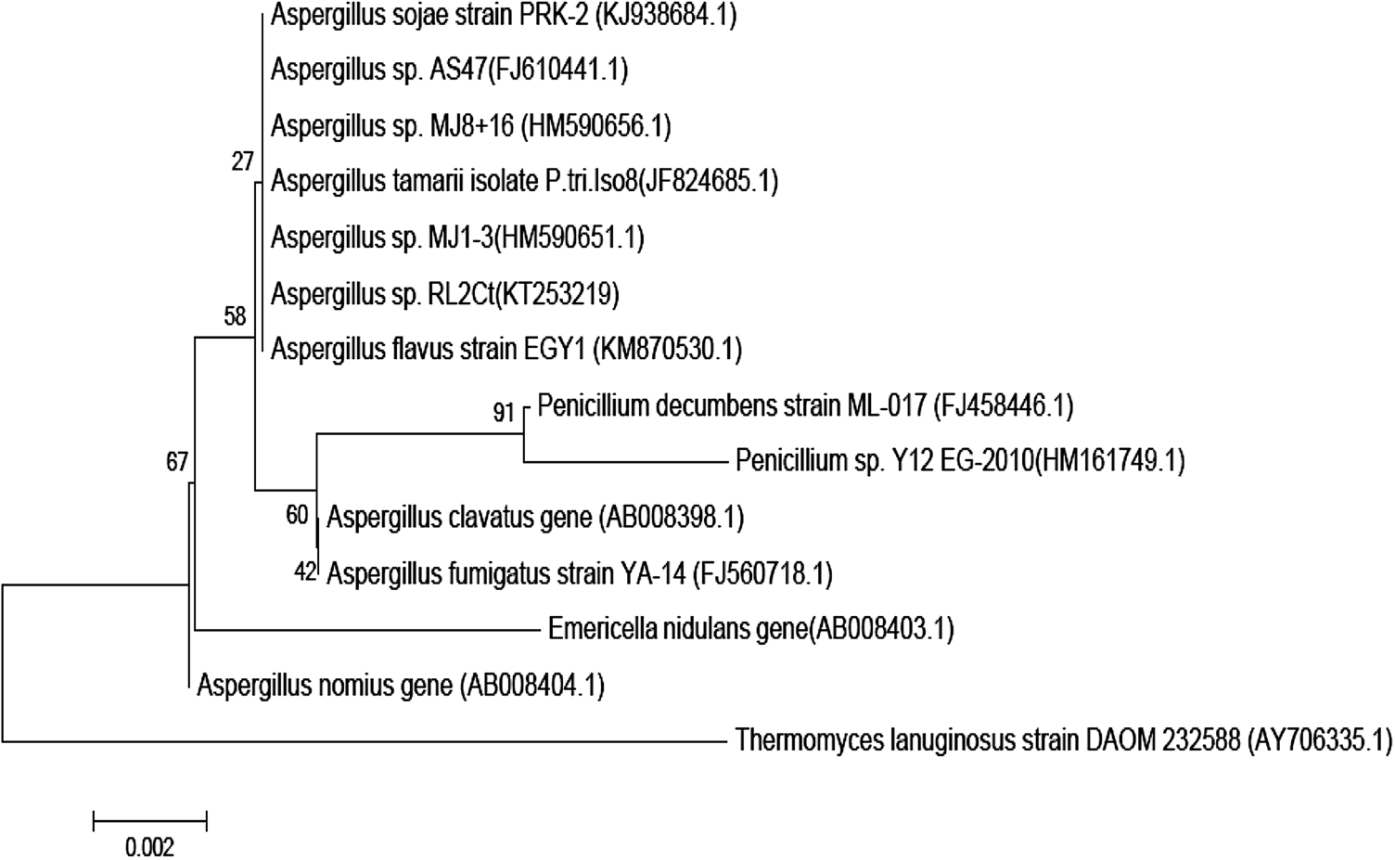
Phylogenetic dendrogram based on the 18S rDNA sequence of *Aspergillus* sp. RL2Ct. Number in parenthesis are accession numbers of published sequences. Bootstrap values were based on 1000 replicates

The 18S rDNA gene sequence of this fungal isolate was carried out and the obtained sequence was deposited in the GenBank database with accession no. KT253219. BLAST analysis of isolate RL2Ct 18S rDNA gene sequence showed 99 % homology with the 18S rDNA sequence of various species of the genus *Aspergillus*. A phylogenetic tree of this sequence was constructed by neighbor joining method with 1000 bootstrap replicates using MEGA version 5.2 (Fig. 3). The result of this phylogenetic analysis was consistent with that of the phenotypic tests. Therefore, isolate RL2Ct was identified as a strain of *Aspergillus* sp. and named *Aspergillus* sp. RL2Ct.

### Optimization of culture conditions

#### Medium optimization

*Aspergillus* sp. RL2Ct was cultured in five different media viz. M1, M2, M3, M4 and M5; maximum production of cutinase (164 ± 4.50 U/mg protein) was observed in M1 medium. The biomass yield was good in M3 and M4 media, but cutinase activity was not recorded. Media M3 and M4 were supplemented with the glucose and yeast extract as source of carbon, which promoted the growth of fungal mycelium but inhibited the production of inducible enzyme. In medium M2, the biomass of *Aspergillus* sp. RL2Ct was fairly good and it exhibited 52 ± 1.63 U/mg protein cutinase activity, but it was less in comparison to M1 medium (Table 1).

**Table 1:** Production of cutinase by *Aspergillus* sp. RL2Ct in various media

Media code	Specific activity (U/mg protein)
M1	164 ± 4.50
M2	52 ± 1.63
M3	00 ± 00
M4	00 ± 00
M5	00 ± 00

#### Screening of carbon and nitrogen sources

A number of carbon and nitrogen sources were tested to enhance growth and cutinase activity of *Aspergillus* sp. RL2Ct. All carbon and nitrogen sources except cutin-containing medium did not exhibit cutinase activity which indicated the inducible nature of *Aspergillus* sp. RL2Ct cutinase. Among various cutin sources used, minimal salt medium containing orange cutin showed maximum cutinase activity (261 ± 4.90 U/mg protein). However, medium containing papaya (164 ± 4.50 U/mg protein) and apple (86 ± 2.64 U/mg protein) cutin exhibited considerable cutinase activity, but much less than the medium having orange cutin (Fig. 1). Hence orange cutin acted as very good carbon source as well as inducer for the induction of cutinase of *Aspergillus* sp. RL2Ct.

#### Screening of important nutrients component using Plackett–Burman design (experimental design technique)

**Fig. 4:**
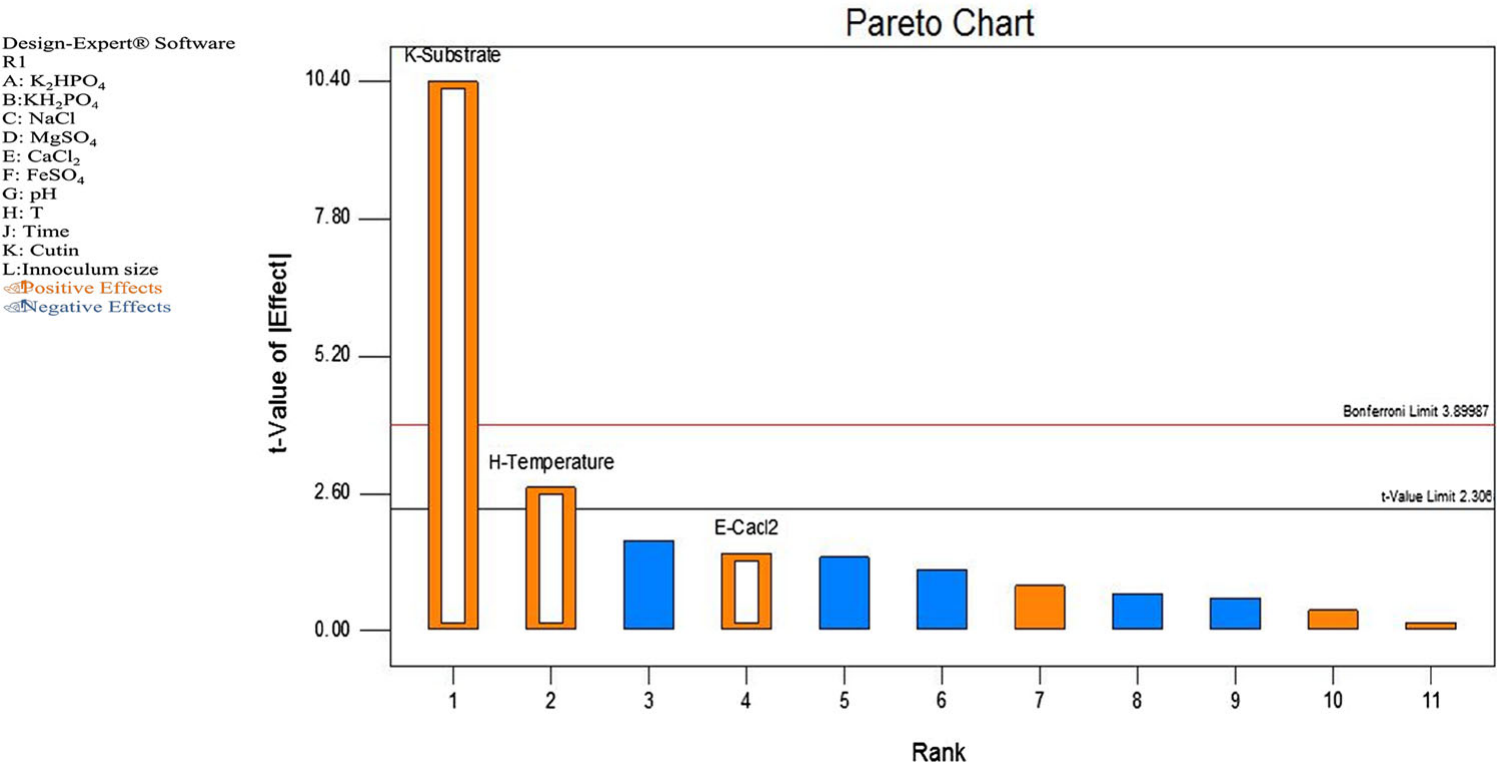
Plackett–Burman design showing the effect of different factors on cutinase production by *Aspergillus* sp. RL2Ct

The effect of 11 independent variables (pH, temperature, time, inoculum, K_2_HPO_4_, KH_2_PO_4_, MgSO_4_, NaCl, FeSO_4_, CaCl_2_ and cutin) was observed on the production of cutinase enzyme and study was carried in 12 runs using Plackett–Burman design. Table 2 shows the 11 selected independent variables and their corresponding response on the production of cutinase activity. A set of 12 experiments exhibited variation in activity ranging from 0 to 64 U/mg protein inferring that the strong influence of medium components on the production of cutinase. Result obtained by performing Plackett–Burman design was further used to construct a Pareto chart (Fig. 4) to find out the order of significance of variables on cutinase production.

**Table 2:** Plackett–Burman experimental design for evaluating the influence of various independent variables on cutinase production by *Aspergillus* sp. RL2Ct

Run	KH_2_PO_4_ (%)	K_2_HPO_4_ (%)	NaCl (%)	MgSO_4_ (%)	CaCl_2_ (%)	FeSO_4_ (%)	pH	T (°C)	Incubation time	Cutin (%)	Inoculum (ml)^a^	Response (U/mg protein)
1	0.05	3	0.5	0.4	0.003	0.01	6	40	18	0.5	2	54
2	1	1	0.5	0.4	0.05	0.01	6	25	36	0	2	0
3	1	3	0.5	0.02	0.003	0.01	8	25	36	0.5	0.5	27
4	1	3	0.01	0.02	0.003	0.05	6	40	36	0	2	0
5	1	1	0.5	0.4	0.003	0.05	8	40	18	0	0.5	0
6	1	1	0.01	0.02	0.05	0.01	8	40	18	0.5	2	51
7	0.05	1	0.5	0.02	0.05	0.05	6	40	36	0.5	0.5	64
8	0.05	3	0.01	0.4	0.05	0.01	8	40	36	0	0.5	0
9	1	3	0.01	0.4	0.05	0.05	6	25	18	0.5	0.5	38
10	0.05	1	0.01	0.4	0.003	0.05	8	25	36	0.5	2	34
11	0.05	1	0.01	0.02	0.003	0.01	6	25	18	0	0.5	0
12	0.05	3	0.5	0.02	0.05	0.05	8	25	18	0	2	0

^a^1 ml of inoculum contains 2.11 × 10^7^ spores

#### Central composite design (CCD) and validation of statistical model

For the determination of optimum level and combined effect of different variables (cutin, temperature and CaCl_2_), a central composite design as given in Table 3 was used and a second-order polynomial equation was derived to explain the dependence of cutinase production on medium components. The results of CCD were fitted into second-order polynomial equation for the prediction of response on the bases of coded value.

$$\begin{aligned} {\text{Responses}} = 671.98 + 11.78A - 7.00B + 18.45C - 3.75AB + 3.75AC \hfill \\ - 7.50BC - 231.99A^{2} - 235.71B^{2} - 186.74C^{2} \hfill \\ \end{aligned}$$

**Table 3:** Central composite experimental design using factors having positive effect on production of cutinase by *Aspergillus* sp. RL2Ct

Run	Cutin (%)	T (°C)	CaCl_2_ (%)	Response (U/mg protein)
1	0.67	35	0.050	60
2	0.50	20	0.100	45
3	0.00	50	0.100	0
4	0.25	09	0.050	39
5	0.25	35	0.133	224
6	0.00	20	0.100	0
7	0.25	35	0.050	267
8	0.50	50	0.001	15
9	0.50	20	0.001	0
10	0.25	35	0.050	560
11	0.25	35	−0.032	92
12	0.50	50	0.100	0
13	0.25	35	0.050	1120
14	0.25	35	0.050	440
15	0.25	35	0.050	860
16	0.25	35	0.050	780
17	0.25	60	0.050	0
18	0.00	50	0.001	0
19	−0.17	35	0.050	0
20	0.00	20	0.001	0

**Fig. 5:**
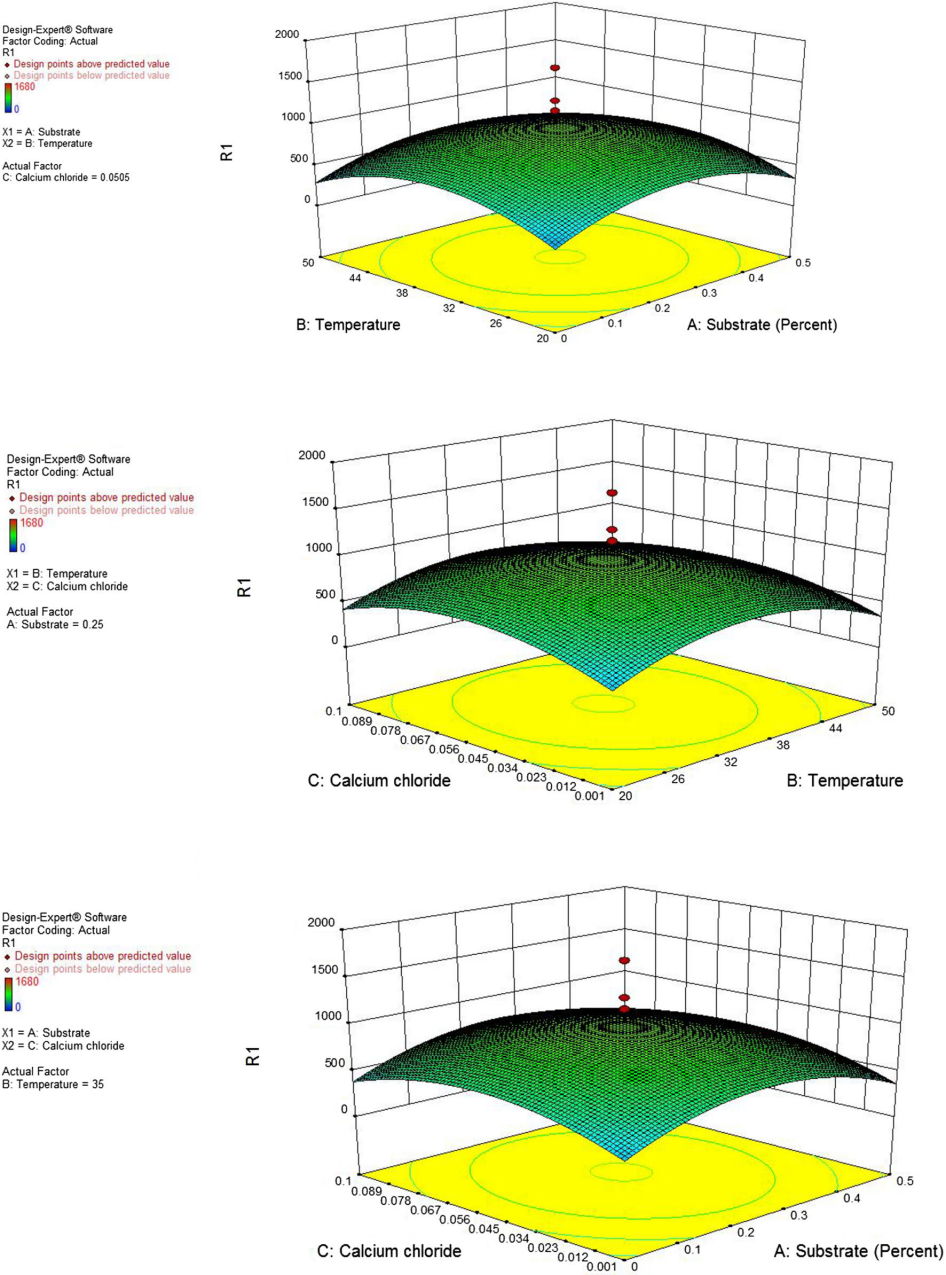
Three-dimensional response surface plots for the effect of **a** *A* substrate (cutin) and *B* temperature. **b** *B* temperature and *C* CaCl_2_. **c** *A* substrate (cutin) and *C* CaCl_2_ on cutinase production by *Aspergillus* sp. RL2Ct

ANOVA analysis of the CCD result was performed and four process orders were suggested by Design expert 9.0. Quadratic and cubic process order gives comparable results but quadratic process order was taken for further analysis due to low standard deviation (220.3) for further analysis (Table 4). The analysis of variance of the quadratic regression model suggested that the model was significant as was evident from correlation coefficient (0.78) which exhibited that the experiments performed were quite reliable. Three-dimensional (3-D) graphs were generated for regression analysis of CCD design, using pair wise combination for cutinase production. These 3-D response surface plots describe the effects of the independent variables and combined effect of each independent variable upon the response (Fig. 5a–c). Interaction effects of coefficient term between cutin, temperature and CaCl_2_ have also been studied. In order to determine the optimal levels of each variable for maximum cutinase production, 3-D response surface plots were constructed by plotting the response (cutinase activity) on the *Z*-axis against any two independent variables, while maintaining other variables at their optimal levels. As shown in Fig. 5a, a curvature in the response surface indicates lower and higher values of both, substrate (cutin) and temperature did not result in higher response. The increment of cutin concentration from 0.1 to 0.25 % and temperature from 25 to 35 °C increased the cutinase activity, but further increment in both the components decreased the enzyme activity (Fig. 5a). A similar profile was observed in Fig. 5b (effect of temperature and CaCl_2_) and Fig. 5c (effect of substrate and CaCl_2_), where cutinase activity increased with increasing temperature up to 35 °C and CaCl_2_ concentration from 0.05 to 0.5 g/l.

**Table 4:** Lack of fit test of CCD calculated value for selection of effective model

Source	Std.	*R* ^2^	Adjusted *R* ^2^	Predicted *R* ^2^	Press	
Linear	373.02	0.0032	−0.1837	−0.2899	2.881E + 006	
2FI	413.76	0.0035	−0.4564	−1.3212	5.184E + 006	
Quadratic	**220.30**	**0.7827**	**0.5872**	**0.6672**	**7.433E** **+** **005**	**Suggested**
Cubic	282.88	0.7850	0.3193	0.4926	1.133E + 006	Aliased

### Validation of model

**Fig. 6:**
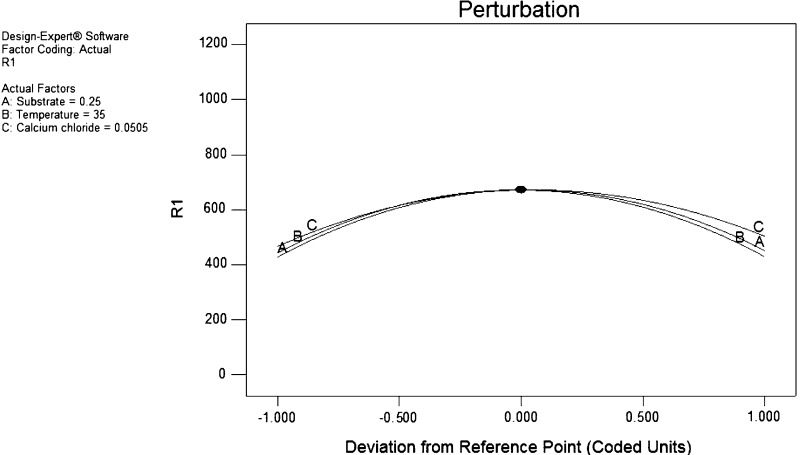
Perturbation plot of three positive variables, i.e., substrate (cutin), temperature and CaCl_2_ on response R1 (here R1 is the enzyme activity expressed in units per milligram protein)

The maximum activity obtained by performing experiment was 663 U/mg protein, which was closely related to the predicted value, i.e., 671 U/mg protein calculated by ANOVA analysis. Perturbation plot (Fig. 6) showed the optimum value for variable temperature 35 °C, substrate (orange cutin) 0.25 g and CaCl_2_ 0.05 g per 100 ml. The model was validated by performing the experiment under optimum condition which resulted in 663 U/mg protein activity, which proves the validity of the model. By performing factorial design, there was 2.5-fold increase in the production of cutinase which reflects the significance of optimization of process parameters.

## Discussion

The present study envisages isolation and screening of cutinase-producing fungi and optimization of culture conditions for cutinase production. Morphological studies, spore staining and 18S rDNA analysis of selected isolate showed that it belonged to the genus *Aspergillus* and named as *Aspergillus* sp. RL2Ct. Different species of this genus, i.e., *A. niger* (Nyyssola et al. 2013), *A. nidulans* (Castroochoa et al. 2012), *A. oryzae* (Liu et al. 2009) have been reported to harbor cutinase activity. Previously cutinases have been isolated from various microorganisms including bacteria (Purdy and Kolattukudy 1975; Chen et al. 2007) and fungi (Fett et al. 1992, 1999).

To harness the maximal potential of a microorganism for the synthesis of desired metabolite/product, it is very important to optimize the cultural parameters. Cultivation of *Aspergillus* sp. RL2Ct in medium M1 resulted in increased cutinase production (164 ± 4.5 U/mg protein) as compared to initial screening media used for the assessment of cutinase activity (153 ± 3.0 U/mg protein). Further increase in cutinase production to 261 ± 3.0 U/mg protein was observed when medium was supplemented with orange cutin. Earlier apple cutin was used for the production of extracellular cutinase in *F. solani pisi*. (Purdy and Kolattukudy 1975; Degani et al. 2006), whereas tomato cutin was used for cutinase production in *Pseudomonas cepacia* NRRL B-2320 (Fett et al. 1992). Most of the reported cutinases showed highest cutinase production in minimal medium supplied with cutin (Chen et al. 2013). Cutin was used as a carbon source or an inducer for achieving high level of cutinase yield (Chen et al. 2007; Fett et al. 2000; Lin and Kolattukudy 1978). Without supplementation of cutin in culture medium, this fungus did not show cutinase activity which indicated inducible nature of its cutinase.

The production of cutinase is influenced by the type and concentration of carbon and nitrogen sources, culture pH, temperature and dissolved oxygen concentration (Du et al. 2007). Different operational variables interact and influence their respective effect on response, thus it is worthwhile to use an experimental design that could account for these interactions (Coninck et al. 2004; Dasu and Panda 2000; Kumar et al. 2009). Cutinase production was the result of a synergistic combination of all the parameters on the microorganism in the culture. Therefore, optimization of cultural conditions was done using response surface methodology. An overall 4.33-fold increase in the production of cutinase was observed after optimization of culture conditions (including 2.5-fold increase using RSM). The formulation of the culture medium using experimental planning, the best results were obtained for cutinase production (663 U/mg protein) in medium containing: KH_2_PO_4_ 0.5 g, K_2_HPO_4_ 2.5 g, NaCl 0.1 g, MgSO_4_ 0.2 g, CaCl_2_ 0.5 g, FeSO_4_ 0.03 g, cutin 2.5 g/l with pH 7.0 and temperature 35 °C, inoculated with an aliquot of 2.11 × 10^7^ spores/ml for 24 h. Most of the cutinase-producing fungal strains i.e., *T. vulgaris*, *T. fusca*, *S. badius, F. oxysporium* had longer production time of 96, 168, 336 and 72 h, respectively (Chen et al. 2013). Calcium chloride was observed as one of the significant factor in medium formulation, as it leads to highly branched hyphae and numerous bulbous cells in *Aspergillus* sp. (Pera and Callieri 1997). Earlier, central composite design has been applied to optimize *P. cepacia* NRRL B 2320 cutinase which resulted in twofold increase in enzyme activity, i.e., 336 µmol/min/ml (Dutta et al. 2013). Cutinase production by *Fusarium oxysporum* also showed twofold (22.68 µmol/min/ml) increase in enzyme activity when optimized using RSM-based design with flax seed oil used as a substrate (Dutta et al. 2013).

Cutinases are important enzymes, but there are some limiting factors in their production e.g., unavailability of commercial pure cutin, which is used as both substrate and inducer for the production of these enzymes.

## Conclusions

The present investigation had led to the isolation of a fungal source of cutinase, i.e., *Aspergillus* sp. RL2Ct with a very high activity for the cleavage of ester linkage. The maximum cutinase activity (663 U/mg protein) was observed in the presence of cutin as an inducer at pH 7 and temperature 35 °C, when inoculated with an aliquot of 2.11 × 10^7^ spores/ml after optimization of culture parameter using RSM. These results revealed that *Aspergillus* sp. RL2Ct is good source of cutinase and its hydrolytic activity can be exploited for industrial application.
